# Soluble Aβ Oligomers Formed Channels Leading to Calcium Dysregulation

**DOI:** 10.1093/function/zqad037

**Published:** 2023-07-17

**Authors:** Shaomin Li

**Affiliations:** Ann Romney Center for Neurologic Diseases, Brigham and Women’s Hospital and Harvard Medical School, Boston, MA 02115, USA

## A perspective on “Endogenous Amyloid-formed Ca^2+^-permeable Channels in Aged 3xTg AD Mice”

Alzheimer’s disease (AD) is a prevalent global neurodegenerative disorder that progressively impairs cognitive function, memory, and behavioral and mood patterns. Accumulation of various forms of Aβ oligomers is considered a significant factor in AD progression. Recent FDA-approved anti-amyloid drugs (Aducanumab and Lecanemab) selectively target amyloid aggregates. The calcium dysregulation hypothesis suggests that abnormal handling of Ca^2+^ in neurons contributes to AD’s development.^1,2^ Disruptions in calcium homeostasis lead to neuronal dysfunction, synaptic damage, and death. Evidence points to the involvement of calcium-related receptors in AD. These receptors include N-Methyl-D-Aspartate (NMDA) receptors, voltage-gated calcium channels (VGCCs), and the upregulation of ryanodine receptors and inositol triphosphate receptors.

In this journal, Li et al. (2023)^3^ revealed that endogenous Aβ can form Ca^2+^-permeable channels in aged 3xTg AD mice. By employing patch-clamp techniques, the authors observed spontaneous Ca^2+^ oscillations in pancreatic acinar cells of aged 3xTg AD mice, where VGCCs are not typically present, while no such oscillations were seen in age-matched wild-type mice. Through the utilization of perforated patch recording, it was demonstrated that the application of Aβ in the recording electrode can effectively permeate the cellular membrane and create a pore on dissociated adult acinar cells. Interestingly, they discovered that the Ca^2+^ oscillations can be eliminated by removing extracellular Ca^2+^, adding ZnCl_2_, or using the Aβ channel blocker Anle138b. Their subsequent investigation revealed that these spontaneous Ca^2+^ oscillations were mediated through Ca^2+^-induced Ca^2+^ release (CICR) rather than InsP3-induced Ca^2+^ release. Notably, these oscillations differed from acetylcholine (ACh)-induced spontaneous Ca^2+^ oscillations. In contrast to previous reports where high concentrations of exogenous Aβ were used to form Aβ channels or pores in vitro, this study suggests the presence of endogenously mediated Aβ channels.

To investigate the formation of Ca^2+^-permeable channels by Aβ, the authors made a prudent decision by selecting pancreatic acinar cells, which typically lack VGCCs but accumulate Aβ deposition, similar to the hippocampus. However, it is worth noting that hippocampal neurons have numerous VGCCs. To eliminate the possibility of intracellular Ca^2+^ elevation due to digestive enzyme activity in pancreatic acinar cells, the authors conducted additional experiments on ACh, a common receptor for secretion in these cells. Interestingly, they observed that the specific Ca^2+^ oscillations mentioned earlier were not affected by atropine, an ACh receptor antagonist. The Ca^2+^ oscillations induced by ACh were completely blocked by the same concentration of atropine. Of great significance, the Ca^2+^ oscillations induced by Aβ (both endogenous and exogenous) are markedly distinct from ACh-induced oscillations. The Aβ-induced oscillations necessitate extracellular Ca^2+^ influx and elicit CICR. Conversely, the ACh-induced oscillations are unaffected by extracellular Ca^2+^ influx and Anle138b. Thus, the initiation and pharmacological response of Aβ-induced Ca^2+^ oscillations differ from those of ACh-induced Ca^2+^ oscillations.

**Figure 1.**
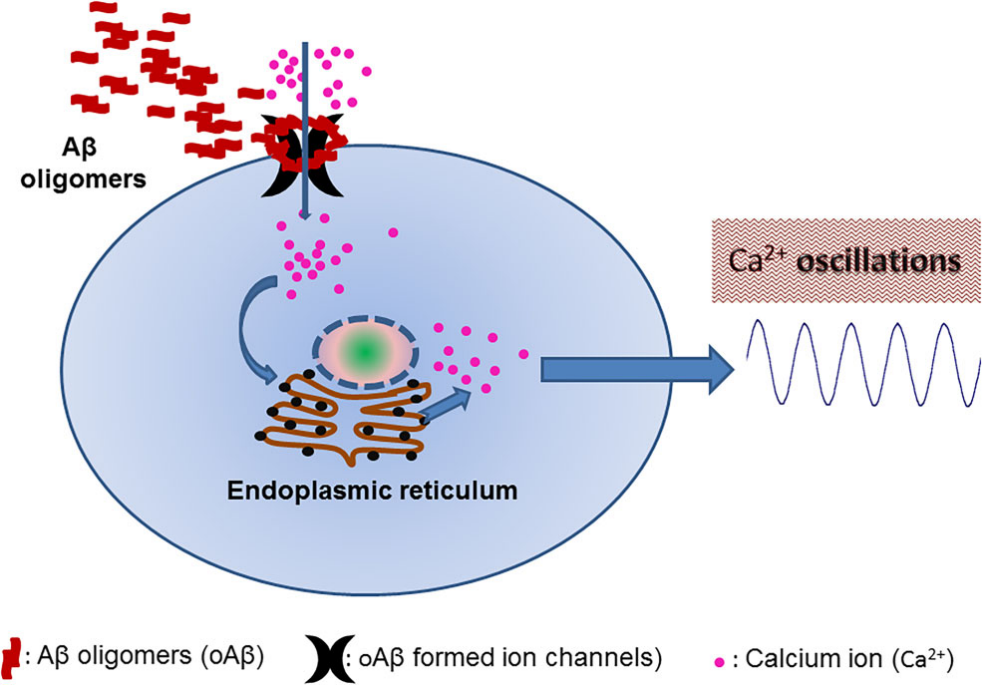
Schematic of the principal pathways identified for endogenous Aβ-formed Ca^2+^-permeable channels. Aβ oligomers engage with the cell membrane and create “ion pores,” facilitating the entry of extracellular Ca^2+^ into the cell. These Ca^2+^ ions, in turn, trigger additional Ca^2+^ release from the endoplasmic reticulum, leading to an increase in intracellular Ca^2+^ levels. This rise in Ca^2+^ levels results in an observable oscillation wave that can be recorded using patch clamp techniques.

It is widely accepted that the primary toxic species affecting brain cells are the soluble Aβ oligomers, instead of the monomers or plaques. These Aβ oligomers exert their influence by interacting with their partners on cell membranes, leading to dysfunction in various membrane proteins (such as cellular prion protein, NMDAR, AMPAR, mGluRs, α7nAChR, the receptor for advanced glycation endproducts and insulin receptor).^4,5^ Several studies have also revealed that the Aβ oligomers possess the ability to create ion channel pores known as Aβ ion channels.^6^ Previous studies on Aβ ion channels have primarily focused on in vitro experiments involving both artificial and natural membranes, wherein high concentrations (ranging from 2 to 15 µm) of soluble Aβ were employed. A recent review has comprehensively outlined the process of Aβ ion channel pore formation based on cutting-edge techniques such as cryo-electron tomography imaging, transmission electron microscopy, and atomic force microscopy.^7^ According to the findings, two distinct types of pores are formed by Aβ oligomers. Firstly, Aβ annular oligomers are seen to insert into the membrane, consequently forming ion channel pores. Secondly, there are Aβ curvilinear protofibrils and oligomers that spread out and embed themselves within the bilayer, leading to membrane permeability and lipid extraction akin to a detergent-like effect. The formation of Aβ ion channels facilitates the influx of Ca^2+^ ions, which in turn triggers the release of intracellularly stored Ca^2+^. Consequently, this disturbance in Ca^2+^ homeostasis leads to imbalances and aberrant activity within the signaling pathways, leading to perturbations in cellular function (Figure 1).

It is important to note that in the current study, Anle138b was used as a blocker for Aβ ion channels,^8^ whereas earlier studies utilized tromethamine and aluminum to block Aβ-induced Ca^2+^ channels.^9^ As of now, there are no approved drugs or compounds specifically designed as Aβ channel blockers for clinical use. However, Anle138b, a small organic molecule, has exhibited promising results in preclinical studies involving animal models of neurodegenerative diseases, including AD. It has been investigated for its potential to inhibit the aggregation and spread of misfolded proteins, such as α-synuclein in Parkinson’s disease and prions in prion diseases. Although Anle138b primarily targets protein aggregation and propagation, it does not selectively block Aβ channels. Its mechanism of action involves stabilizing the structure of misfolded proteins and preventing their harmful effects. By combining voltage or current protocols with well-known ion channel blockers and applying them to the membrane patch, different types of ion channels can be selectively activated. This allows for the specific measurement of their activity, facilitating the detection of Aβ-induced Ca^2+^ channels.

In the present study, Li et al.^3^ discovered that endogenous Aβ oligomers possess the capability to create a Ca^2+^ channel, thereby disrupting the delicate balance of Ca^2+^ levels within cells. This finding contributes to our comprehension of the existing literature on the hypothesis of calcium dysregulation^1,2^ and the role of Aβ-interact with membrane proteins^4,5^ in the etiology of AD. Further examination of the characteristics and mechanisms of Aβ-generated Ca^2+^ channels in neurons could yield significant insights into the molecular mechanisms underlying AD and its associated pathophysiological features. Consequently, this investigation establishes a foundation for future research investigating the impact of Aβ on Ca^2+^ functionality in animal models of AD, with the expectation that such studies will illuminate potential therapeutic targets for the effective treatment of AD.

## Supplementary Material

zqad037_Supplemental_File
