# Supplementary material for: Soluble Aβ Oligomers Formed Channels Leading to Calcium Dysregulation
Source: Function (Oxf). 2023 Jul 17;4(5):zqad037. doi: 10.1093/function/zqad037 (PMC10423025; doi:10.1093/function/zqad037)
Supplement: zqad037_Supplemental_File [file zqad037_supplemental_file.docx]

**Soluble Aβ oligomers formed channels leading to calcium dysregulation**

A perspective on 'Endogenous amyloid-formed Ca^2+^-permeable channels in aged 3XTg AD mice'

Shaomin Li^1^

^1^Ann Romney Center for Neurologic Diseases, Brigham and Women's Hospital and Harvard Medical School, Boston, MA 02115, USA

**Correspondence to:**

Dr. Shaomin Li,

Ann Romney Center for Neurologic Diseases, Brigham and Women’s Hospital and Harvard Medical School, 60 Fenwood Road, Boston. MA 02115. USA.

Tel: 617-5255119

E-mails: [sli11@bwh.harvard.edu](mailto:sli11@bwh.harvard.edu)
